# Eco-Mechanical Optimization of Composite-Amended Sandy Substrate for *Alhagi sparsifolia* in Arid Regions

**DOI:** 10.3390/plants15040605

**Published:** 2026-02-14

**Authors:** Meixue Zhang, Qinglin Li, Xiaofei Yang, Penghu Feng, Wenjuan Chen, Guang Yang

**Affiliations:** 1 College of Water Resources and Construction Engineering, Shihezi University, Shihezi 832000, China; 2 Key Laboratory of Cold and Arid Regions Eco-Hydraulic Engineering of Xinjiang Production & Construction Corps, Shihezi 832000, China; 3 College of Science, Shihezi University, Shihezi 832000, China

**Keywords:** *Alhagi sparsifolia*, trait plasticity, root sand fixation, root–soil composite shear strength, arid-region slope ecological restoration

## Abstract

In response to the problems of loose soil structure and insufficient water and nutrient retention capacity of sandy bank slopes in arid regions, which constrain vegetation establishment and long-term slope stability, this study focuses on typical sandy soils in arid northwestern China. The desert plant *Alhagi sparsifolia*, characterized by clonal root sucker reproduction, was selected as the study species to construct and optimize a composite-amended sandy substrate suitable for ecological restoration of bank slopes. Based on an orthogonal experimental design, carboxymethyl cellulose sodium (CMC), straw fibers (SF), and fly ash (FA) were combined at different proportions to assess (i) the vertical distribution of soil water and nutrients in the *A. sparsifolia* growth habitat, (ii) aggregate structure, (iii) plant trait responses to environmental regulation, and (iv) the shear strength of root–soil composites. The results indicate that when the contents of CMC, SF, and FA were 0.5%, 1.0%, and 5.0%, respectively, the substrate environment promoted a vertically oriented root system with pronounced lateral root development in *A. sparsifolia*, and the plants adopted an adaptive strategy that balances resource acquisition efficiency and environmental constraints by regulating aboveground growth allocation. This growth pattern reduced the risk of disturbances to slope stability caused by excessive aboveground biomass while maintaining the sand-fixing function of root morphological traits. This study provides a plant functional trait-based regulation strategy for ecological restoration of typical sandy slopes in arid regions, and the proposed composite substrate optimization scheme offers a feasible reference for improving vegetation establishment and substrate performance in sandy habitats.

## 1. Introduction

In the arid regions of northwestern China, the construction of mountainous reservoirs provides the main water source for downstream urban agglomerations, rural villages, and agricultural irrigation areas [1,2], but the changes they induce in downstream river hydrological processes have led to significant degradation of riparian vegetation, increasing regional ecological security risks [3].

Sandy soil is a typical soil type of riverbank slopes in northwestern China and is characterized by low clay content, high permeability, and a loose and easily fragmented structure [4,5]. Coupled with the arid natural environment of northwestern China, soils with low water content exhibit insufficient shear strength, which more easily triggers slope instability and wind erosion damage, seriously threatening the safety of riverbank infrastructure and the sustainable development of the ecological environment [6,7]. Meanwhile, desert vegetation has gradually become a research focus in related scientific studies and engineering applications due to its low ecological water requirement and well-developed root systems [8,9]. A large number of studies have demonstrated the positive role of desert vegetation in slope stability and ecological restoration in arid regions [10,11]. Using the perennial phreatophyte sub-shrub leguminous plant *Alhagi sparsifolia* (Fabaceae; sparse leaved camelthorn or camelthorn) as an example, it is characterized by a low plant height (approximately 25–40 cm) and a well-developed root system (up to 20 m in length), and it exhibits strong drought tolerance, salt tolerance, and stress resistance [12]. Flora of China provides detailed morphological descriptions and illustration information for this species [13]. Relevant studies have also confirmed its strong soil-fixing and slope-protection capacity [14,15].

However, in practical applications, desert vegetation also faces prominent challenges. For example, *A. sparsifolia* shows extremely limited natural seedling recruitment. Even when floods or rainfall trigger germination [3], successful establishment is rare, and the species mainly reproduces clonally through root suckers [16,17]. Related studies have indicated that *A. sparsifolia*, as an important mycorrhizal symbiont in desert ecosystems, adapts to environmental stress by establishing symbiotic relationships with soil microorganisms [18,19]. Recent studies have further demonstrated that its seedlings can form an efficient symbiotic structure with arbuscular mycorrhizal fungi (AMF), effectively promoting seedlings to rapidly pass through the vulnerable growth stage under drought stress [20]. Researchers have confirmed the significant association between AMF and the desert riparian plant *A. sparsifolia*, and elucidated its reproductive strategy of in vivo regeneration under harsh conditions.

The above findings indicate that the establishment and persistence of desert vegetation are highly dependent on habitat conditions. Therefore, engineering measures to improve the growth substrate are crucial for the ecological restoration on sandy riparian bank slopes. Vegetation Concrete (VC), as a typical artificial composite ecological substrate, provides a favorable environment for vegetation establishment [21] and shows significant advantages in shallow slope protection that is prone to water flow scouring, such as along rivers, lakes, and reservoir water-level fluctuation zones [22]. Sandy clay loam with favorable water, nutrient, air, and thermal conditions is the preferred soil material for preparing vegetation concrete [23]. However, in arid regions dominated by sandy soil and silty soil, ecological substrates formulated from these soils often exhibit weak cohesion, low water and nutrient retention capacity, and poor adaptability to harsh climatic conditions [24], thereby limiting their practical application in ecological slope protection engineering. To ensure the long-term self-maintenance of ecological slope protection systems, sandy substrates should be optimized to facilitate root penetration into the underlying sand layer and improve root-induced soil reinforcement, thereby enhancing slope stability [22]. However, riparian vegetation may also pose risks of negatively affecting bank stability [25]; tall shrubs can disturb riverbank slope stability under the action of wind and water erosion [26,27].

Fly ash, sludge, and straw fibers have attracted widespread attention as sustainable alternative materials and solid-waste resources due to their richness in nutrients and organic matter or their favorable physical properties. Previous studies have confirmed their significant effects in improving soil fertility and soil physicochemical properties [28,29,30]. Furthermore, many researchers have proposed ecological composite schemes based on the synergistic effects of multiple materials and applied them in the field of geotechnical engineering. For example, Li, et al. [31] used fly ash and polyacrylamide to improve coal mine waste soil, greatly enhancing its reclamation potential. The fly ash–polyacrylamide soil improvement technology has also exhibited significant improvement effects in sandy soils with poor structure, low fertility, and weak water-holding capacity, manifested as enhanced resistance to water and wind erosion and improved water retention performance [32,33]. In recent years, the combined application of fibers and bio-gum has also been widely used in slope stabilization to promote slope vegetation restoration. Bu, et al. [34] revealed the roles of sisal fiber and polyacrylamide in improving soil water retention and crack resistance, providing experimental and data support for applications in engineering, soil and water conservation, and geological disaster prevention and control. Yan, et al. [35] confirmed that ecological composite materials composed of bio-gum, artificial fiber, coco coir, and wood wool can effectively reinforce loess, stabilize bare slopes in loess regions, and demonstrate strong potential for ecological restoration.

At present, drylands now cover almost 41% of global land (excluding Antarctica) [36], and ongoing global warming is expected to further expand the extent of arid regions [12]. Against the background of continuous ecosystem degradation, maintaining soil multifunctionality (e.g., soil nutrient retention and soil water regulation) is particularly critical [37]. Despite extensive research on composite amendments, they have been rarely incorporated into sandy-substrate design for establishing desert vegetation in arid regions. In particular, few studies have systematically clarified how composite amendments regulate reconstructed soil habitat conditions, thereby inducing plant trait changes and altering root–soil interactions to enhance the shear strength of root–soil composites. Moreover, the long-term ecological restoration performance and environmental adaptability of such engineered substrates under harsh arid conditions remain insufficiently explored.

This study aims to develop sandy substrates widely applicable to ecological restoration in arid regions by utilizing industrial and agricultural solid waste materials, provide a theoretical basis for the selection of composite improved substrate materials for vegetation slope protection in arid regions, and promote the development of riverbank slope engineering in arid regions from short-term artificial intervention toward long-term natural restoration functions. We hypothesized that (i) the synergistic incorporation of carboxymethyl cellulose sodium (CMC), straw fibers (SF), and fly ash (FA) would improve the ecological performance of sandy substrates, thereby promoting the growth and trait plasticity of *A. sparsifolia*; and (ii) the improved substrate would enhance root penetration into the underlying sandy soil and increase the shear strength of root–soil composites, thereby improving slope stability.

## 2. Materials and Methods

### 2.1. Components of Sandy Substrate

The sandy substrate (hereafter referred to as the substrate) in this study consists of planting soil, cement, peat soil, slow-release fertilizers, water retention agent, straw fibers, and fly ash. The sources, key properties/specifications, and preparation/notes of all materials are summarized in Table 1.

### 2.2. Orthogonal Design

The amount of substrate materials was determined according to the Chinese national industry standard “Technical Specification for Ecological Restoration of Steep Slope Vegetation Concrete in Hydropower Engineering” (NB/T 35082, 2016) [38], with all material quantities based on the dry weight of the planting soil. In this study, the amounts of cement (8%), peat (4.5%), and slow-release fertilizers (0.5%) were fixed, and a three-factor three-level orthogonal design (L_9_3^3^) was used to optimize the proportions of CMC, SF, and FA in the ecological substrate. The factor levels and the orthogonal design matrix are presented in Table 2 and Table 3, respectively, with the substrate without amendments as the control group (CK). The experiment consisted of 9 experimental groups and 1 control group, with 12 repetitions per group.

### 2.3. Field Planting

**Figure 1:**
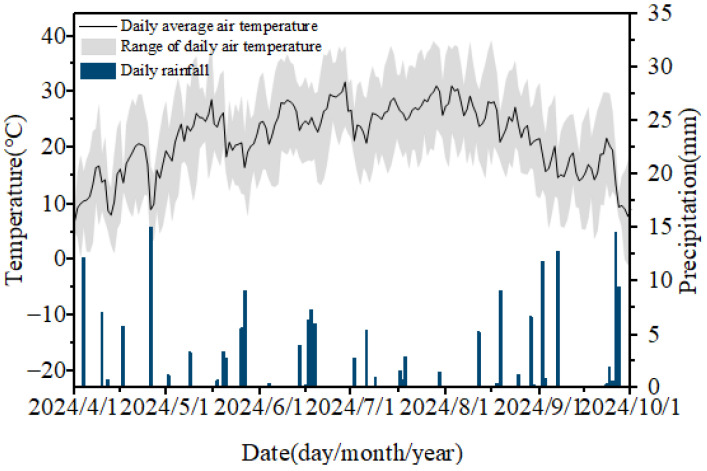
Daily air temperature and precipitation during the experimental period.

The field experiment was conducted from April 2024 to October 2024 at the Water-Saving Irrigation Experimental Station of Shihezi University in Xinjiang, China (44°19′34″ N, 85°59′46″ E). The specific meteorological data during the experiment are shown in Figure 1. The daily average temperature reached 21.8 °C, with very little precipitation, the daily average precipitation being only 1 mm.

The experimental plant was the typical desert species *A. sparsifolia*, which is widely distributed in Xinjiang, China, and exhibits strong drought resistance and salt-alkali tolerance [39,40]. The experimental seeds and planting soil were collected from the same area. Prior to sowing, seeds were soaked in distilled water for 24 h to improve germination [41]. Field planting was conducted using 120 polyvinyl chloride (PVC) pipes (diameter 20 cm, height 55 cm), corresponding to 9 experimental groups and 1 control group with 12 independent replicates per group, which were arranged in a pit (9.8 m × 2.8 m × 0.5 m) with an inter-row spacing of 20 cm and a row spacing of 30 cm. Each PVC pipe was filled with 30 cm of sandy soil at the bottom and 20 cm of substrate at the top, compacted in layers to a dry density of 1.58 g/cm^3^ to provide a growth medium for *A. sparsifolia*. Twenty (20) seeds were sown in each PVC pipe at a depth of 1–2 cm [42]. After sowing, flood irrigation was applied to ensure uniform water distribution [43]. A 5 cm space was reserved at the top of the PVC pipes for installing drip irrigation tapes. During the emergence stage, irrigation was set at 150 mL per pipe per day to ensure seed germination and early growth, and intermittent irrigation was applied thereafter according to plant growth requirements. Once the seedlings stabilized, the excess seedlings were removed to retain one plant per pipe, and plants were grown for 6 months.

### 2.4. Experimental Design and Sample Preparation

#### 2.4.1. Experimental Scheme

**Figure 2:**
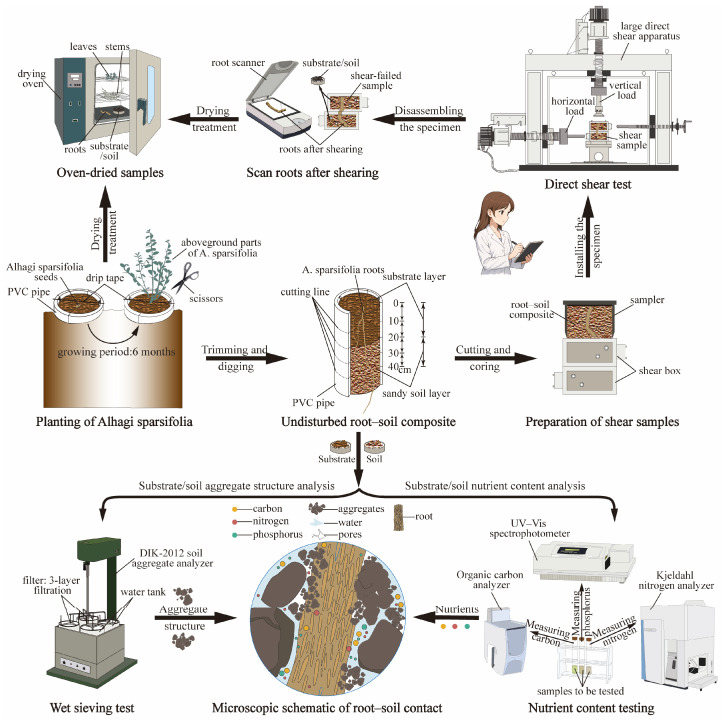
Schematic diagram of the experimental procedure.

All test samples were prepared after 6 months of planting. These samples were used to determine the growth parameters of *A. sparsifolia* as well as the nutrient content, structural characteristics, and mechanical properties of the planting habitat. In this study, the samples used in the experiment were divided into three categories: aboveground parts of *A. sparsifolia*, undisturbed soil samples, and undisturbed root–soil composite samples. The undisturbed root–soil composite samples consisted of the remaining soil columns containing roots after removal of the aboveground plants. The specific parameters and measurement contents of each sample type are presented in Table 4, and the detailed experimental procedure is illustrated in Figure 2.

#### 2.4.2. Plant Samples

To measure the growth indicators of *A. sparsifolia* in the 10 treatment groups, 3 healthy *A. sparsifolia* plants (a total of 30 plants) with consistent growth patterns were selected from each treatment group as standard sample plants. The key growth parameters included plant height, crown width, basal diameter, and number of shoot branches. Subsequently, the aboveground parts of the plants were clipped from the soil surface, and the stems and leaves were separated, with the number of leaves counted.

#### 2.4.3. Soil Samples

To measure soil aggregate stability and nutrient contents, soil cores were collected from the substrate (0–20 cm) and sandy soil (20–40 cm) layers using a custom steel tube (height 5 cm, inner diameter 5 cm). One soil core was taken every 6 cm along the soil profile, yielding 6 soil cores per planting pipe and a total of 180 soil core samples (10 treatments × 3 replicates × 6 samples). For each treatment, the 9 cores from the same layer were composited into one sample and air-dried naturally in the laboratory. After removing impurities, part of each composite sample was broken into aggregates of approximately 1 cm in diameter for wet-sieving analysis, while the remaining part was sieved through a 2 mm mesh and used for soil nutrient determination.

#### 2.4.4. Root–Soil Composite Samples

To obtain root–soil composite samples from different soil depths, root–soil columns were collected after cutting off the aboveground parts and transported to the laboratory. A custom-made square sampler (10 cm in length, 10 cm in width, and 8.5 cm in height) was used to collect root–soil composite samples every 10 cm from top to bottom, with a sampling depth of 40 cm. A total of 4 samples were obtained from each soil column, resulting in 120 samples in total (10 treatments × 3 replicates × 4 samples).

### 2.5. Measurement and Analytical Methods

#### 2.5.1. Measurement of Plant Growth Indicators

The plant height and the crown width in the north–south and east–west directions were measured using a ruler, and the basal diameter was measured using a vernier caliper (with a precision of 0.01 mm). The separated stems and leaves were collected in an iron tray, placed in an oven at 80 °C, and dried for 48 h until a constant weight was reached, then the dry weight was recorded [44].

#### 2.5.2. Measurement of Soil Physicochemical Properties

For the two different soil layers (the substrate layer and the sandy soil layer), 150 g of soil samples were weighed, and soil organic carbon (SOC), total nitrogen (TN), and total phosphorus (TP) contents were determined using the potassium dichromate oxidation–spectrophotometric method, the Kjeldahl method, and the alkali fusion–molybdenum antimony anti-spectrophotometric method, respectively. Detailed measurement procedures followed Soil Agrochemical Analysis (3rd Edition) [45].

The aggregate size distribution was determined using the wet-sieving method [46]. For the two different soil layers, 100 g of soil samples were weighed and placed into an aggregate analyzer (DIK-2012) equipped with sieves of 2 mm, 0.25 mm, and 0.053 mm mesh sizes for wet sieving (oscillation amplitude of 3.8 cm and frequency of 30 times/min). After sieving, the sieve sets were removed, and samples retained on each sieve were washed into aluminum boxes to separate four particle-size fractions: >2.0 mm (large macro-aggregates), 0.25–2.0 mm (small macro-aggregates), 0.053–0.25 mm (micro-aggregates), and <0.053 mm (silt and clay particles). These samples were dried in an oven at 55 °C to constant weight [47], cooled, and weighed. The mass proportions of aggregates in each size fraction were calculated, and water-stable aggregates (WSA) and mean weight diameter (MWD) were used as indicators to evaluate soil aggregate stability [48], as shown in Equations (1)–(3).

(1) $$W_{i}=M_{i}/\sum_{i=1}^{n}M_{i}$$

(2) $$WSA=\sum_{i=1}^{2}W_{i}$$

(3) $$MWD=\sum_{i=1}^{n}X_{i}W_{i}$$

where $W_{i}$ is the mass proportion (%) of the remaining aggregate size fractions on each sieve; $M_{i}$ is the mass (g) of aggregates in the ith size fraction; WSA (water-stable aggregates) represents the proportion (%) of aggregates larger than 0.25 mm; MWD is the mean weight diameter (mm); $\;X_{i}$ is the mean diameter (mm) of aggregates in the ith size fraction; and n is the number of particle-size classes; i= 1, 2, 3, 4, represent the aggregate size > 2.0 mm, 0.25–2.0 mm, 0.053–0.25 mm, and <0.053 mm, respectively.

#### 2.5.3. Direct Shear Test and Shear Strength Indicators

The overall workflow schematic shown in Figure 2 clearly illustrates the key steps of the direct shear test (including specimen preparation, specimen installation, direct shear testing, and sampling), as well as the main components of the direct shear apparatus. In this study, the root–soil composite direct shear test was conducted using the DZJ-10 large-scale frozen soil direct shear apparatus developed by the research team. The direct shear apparatus mainly consists of three main parts: the loading system, sample preparation and shear system, and data collection and control system. The device is equipped with a pressure sensor (with a maximum normal load of 100 kN and a precision of 0.01 kN) and a displacement sensor (with a maximum horizontal displacement of 100 mm and a precision of ±0.1%). The internal dimensions of the shear box are 10 cm × 10 cm × 8.5 cm (length × width × height). After pressing three samples from the same soil depth of each treatment group into the shear box, normal stress values of 50, 100, and 150 kPa were applied respectively, according to the Chinese national standard (GB/T50123-2019) [49], and the samples were sheared at a rate of 1.2 mm/min. The test was concluded when the shear strain reached 20%. When processing the direct shear data, the peak stress was preferentially taken as the strength value. If there was no peak, the strength corresponding to a strain of 20% was taken as the failure strength. The shear strength parameters (cohesion and internal friction angle) of the root–soil composite samples were calculated using Equation (4) [50].

(4) $$\tau_{f}=c+\mathrm{\sigma tan}\varphi$$

where $\tau_{f}$ is the shear strength (kPa); σ is the vertical stress (kPa); φ is the friction angle (°); and c is the cohesion (kPa).

After the shear test, soil samples were collected from the shear plane to determine their water content (dried at 105 °C for 24 h); the roots in the shear samples were then removed and cleaned. Using a root scanner (Phantom 9980XL, D&J (Shanghai) Technology Co., Ltd., Shanghai, China) with a resolution of 600 dpi, the root length (RL), root diameter (RD), root surface area (RSA), and root volume (RV) were measured. After scanning, the roots were placed in an iron tray and dried in an oven at 80 °C for 48 h to determine the dry weight. For each sample from different depth layers, specific root length (SRL), root surface area density (RSAD), and root volume density (RVD) were calculated as follows to further analyze root morphological characteristics:

(5) SRL=RL / RM

(6) RSAD=RSA / V

(7) RVD=RV / V

In the formula, SRL is the specific root length (m·g^−1^); RL is the total root length (m); RM is the root dry weight (g); RSAD is the root surface area density (m^2^·m^−3^); RSA is the root surface area (m^2^); V is the sample volume, equal to 10 × 10 × 8.5 (cm^3^); RVD is the root volume density (dm^3^·m^−3^); RV is the root volume (m^3^).

### 2.6. Statistical Analysis

One-way analysis of variance (ANOVA) and least significant difference (LSD) multiple comparison tests were used to compare soil habitat characteristics and the growth performance of *A. sparsifolia* among treatments. Pearson correlation analysis was employed to examine the relationships among sandy substrate characteristics, plant traits, and the shear strength of the deep (sandy) soil layer; results were visualized using a correlation heatmap. Based on the correlation structure, representative indicators with minimal redundancy were selected for integrated evaluation. All statistical analyses were performed using SPSS version 26.0, and figures were generated using Origin 2024.

## 3. Results

### 3.1. Water and Nutrient Characteristics of Reconstructed Soil Habitats Under Different Amendment Contents

**Figure 3:**
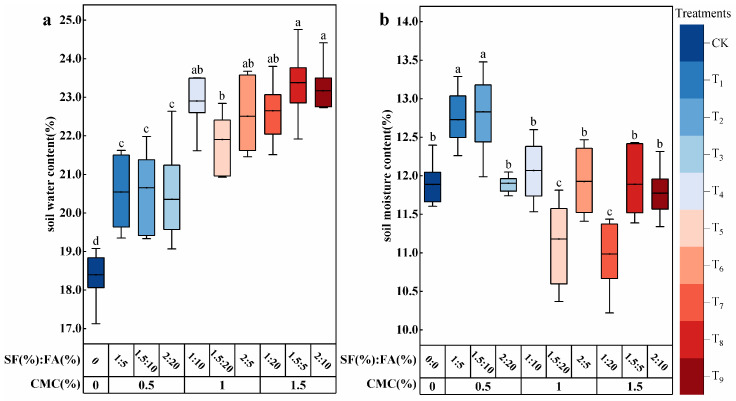
Soil water content in (**a**) the substrate layer (0–20 cm) and (**b**) the sandy soil layer (20–40 cm) under different amendment application rates. Note: the lower and upper boundaries of each box represent the 25th and 75th percentiles, respectively, and the horizontal line inside the box indicates the mean (n = 3). Whiskers extend to 1.5 times the interquartile range. Different lowercase letters above the boxes indicate significant differences among treatments (one-way ANOVA followed by the LSD test, *p* < 0.05). (**a**) represents the variation in soil moisture content within the 0–20 cm substrate layer, and (**b**) represents the variation in soil moisture content within the 20–40 cm sandy soil layer.

Figure 3a shows that the substrate water content in all treatments amended with CMC, SF, and FA was significantly higher than that of CK (one-way ANOVA followed by the LSD test, *p* < 0.05). Overall, substrate water content increased with increasing CMC content, by 10.63–27.10% compared with CK. Under low and medium CMC contents (0.5% and 1.0%), the substrate water content first increased and then decreased with increasing SF/FA ratios, whereas under high CMC content (1.5%), it increased with increasing SF/FA ratios. As shown in Figure 3b, the trend in water content in the 20–40 cm sandy soil layer at the same CMC content was consistent with that in the substrate layer, but the overall water content was significantly lower than that in the 0–20 cm substrate layer. Sandy soil water content showed an overall decreasing trend with increasing CMC content in the substrate layer. Specifically, T_7_ and T_5_ were significantly lower than CK (*p* < 0.05), decreasing by 7.60% and 5.97%, respectively. In contrast, T_1_ and T_2_ were significantly higher than CK (*p* < 0.05), increasing by 7.04% and 7.91%, respectively. This indicates that the composite amendments significantly regulated the soil water status of the reconstructed habitat.

**Figure 4:**
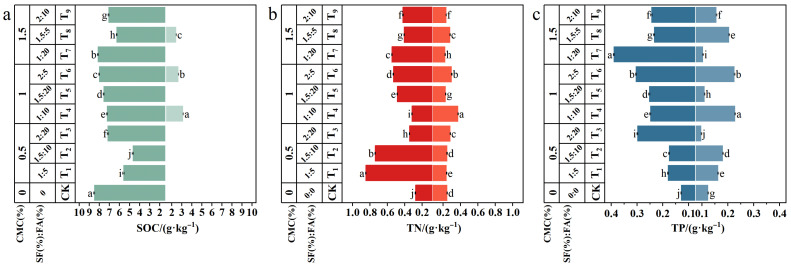
Changes in soil nutrient contents in reconstructed soil habitats under different amendment application rates. Note: (**a**–**c**) represent the contents of soil organic carbon (SOC), total nitrogen (TN), and total phosphorus (TP) in the substrate layer and sand layer under different treatments. For CK and treatments T_1_–T_3_, T_5_, T_7_ and T_9_, SOC contents in the 20–40 cm layer were all below the detection limit; therefore, only T_4_, T_6_, and T_8_ were included in the statistical analysis. Bars represent the mean ± standard deviation (SD, n = 6). Different lowercase letters above the bars indicate significant differences in soil nutrient contents within the same soil layer under different treatments (one-way ANOVA followed by the LSD test, *p* < 0.05).

As shown in Figure 4, SOC, TN, and TP contents decreased with increasing soil depth across all treatments. In the 0–20 cm substrate layer, compared with CK, SOC content in the amended treatments decreased significantly by 4.19–44.99%, whereas TN and TP contents increased significantly by 14.84–205.65% and 36.09–201.91%, respectively (*p* < 0.05). Overall, SOC and TP contents increased with increasing CMC content, while TN content showed an opposite trend. Under low CMC content (0.5%), SOC and TP contents followed the order T_3_ > T_1_ > T_2_, whereas TN content followed T_1_ > T_2_ > T_3_. Under medium and high CMC contents (1% and 1.5%), SOC, TN, and TP showed consistent variation trends among treatments, with the highest nutrient contents observed in T_7_, followed by T_6_, and the lowest in T_8_. In the 20–40 cm sandy soil layer, SOC was detected only in T_4_, T_6_, and T_8_ (detection limit 2.4 g/kg), with values of 3.14, 2.67, and 2.44 g/kg, respectively. The T_4_ treatment exhibited significantly higher TP and TN contents than the other treatments, reaching 0.372 g/kg and 0.229 g/kg, respectively. Analysis indicated that, at the same CMC level, nutrient contents in the sandy soil layer generally followed the same trends as soil water content among treatment groups.

### 3.2. Analysis of Soil Aggregate Stability in Reconstructed Soil Habitats Under Different Amendment Contents

**Figure 5:**
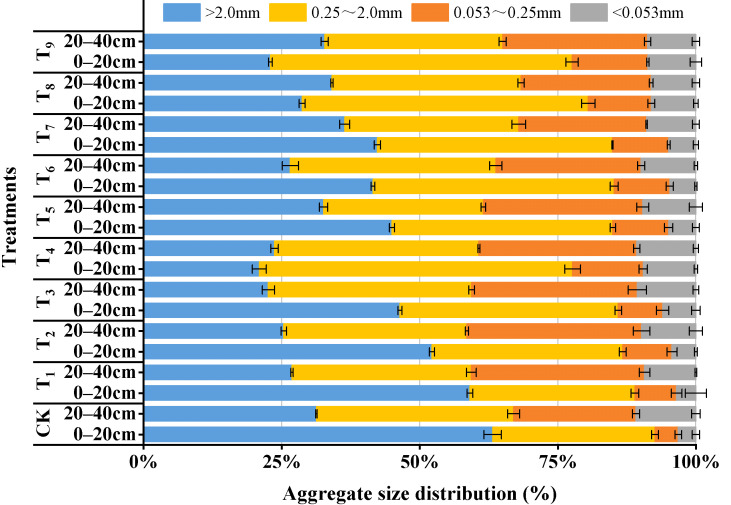
Aggregate size distribution under different treatments.

Figure 5 presents the aggregate size distribution results for each treatment group. In the substrate layer, CK, T_1_–T_3_, and T_6_ showed a sequential decrease in the proportions of large macro-aggregates (>2 mm), small macro-aggregates (0.25–2 mm), micro-aggregates (0.053–0.25 mm), and silt and clay particles (<0.053 mm). In contrast, the other treatments exhibited a pattern in which aggregate fractions first increased and then decreased as particle size decreased. In CK, large macro-aggregates accounted for the highest proportion (63.22%). Overall, the amended treatments were characterized by a reduction in large macro-aggregates and a corresponding increase in finer fractions compared with CK. Compared with the substrate layer, the sandy soil layer showed lower proportions of large macro-aggregates and small macro-aggregates and higher proportions of micro-aggregates and silt and clay particles. No significant differences were observed in the silt and clay particle contents among treatment groups in the sandy soil layer (*p* < 0.05).

As shown in Table 5, WSA in the substrate layer decreased under the amended treatments compared with CK, with relatively higher values in T_1_–T_3_ (85.96–88.98%) and the lowest values observed in the T_8_ (76.09%). In the sandy soil layer, WSA was markedly lower than that in the substrate layer, with the most pronounced reductions observed at the low CMC level (0.5%). Table 5 also lists the MWD results for both soil layers. Significant differences in MWD were observed among treatments in the substrate layer (*p* < 0.05), and the incorporation of amendments generally reduced MWD compared with CK, with the greatest reduction observed in T_8_ (33.45%). Overall, the structural stability of the sandy soil layer was weaker than that of the substrate layer, as reflected by reduced soil aggregate stability indices, particularly in CK and T_1_–T_3_. In summary, the amendments altered aggregate size distribution and stability in the reconstructed habitat, which is linked to erosion resistance and habitat quality.

### 3.3. Aboveground Growth Characteristics of A. sparsifolia Under Different Amendment Contents

**Figure 6:**
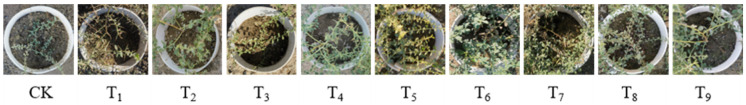
Differences in aboveground growth of *A. sparsifolia* under different treatments.

**Figure 7:**
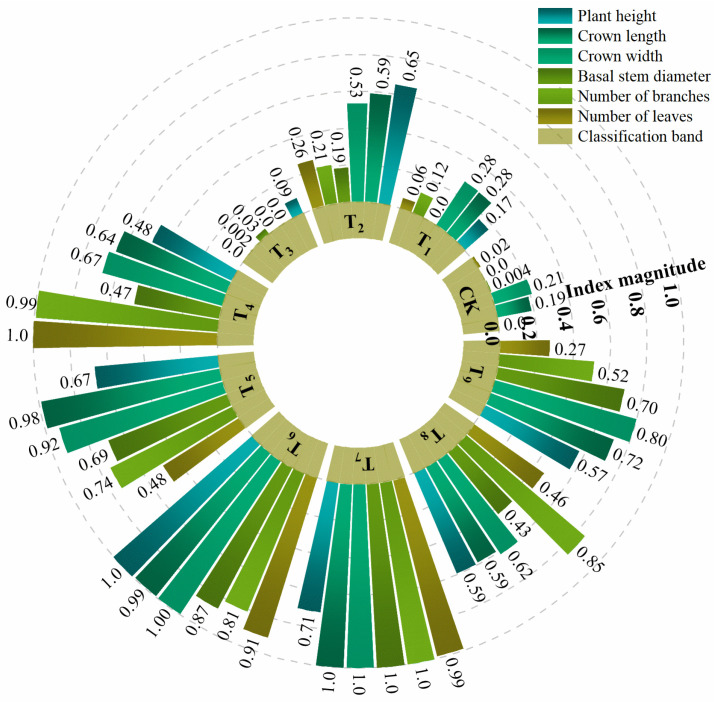
Variations in aboveground plant parameters of different treatments (normalized).

**Figure 8:**
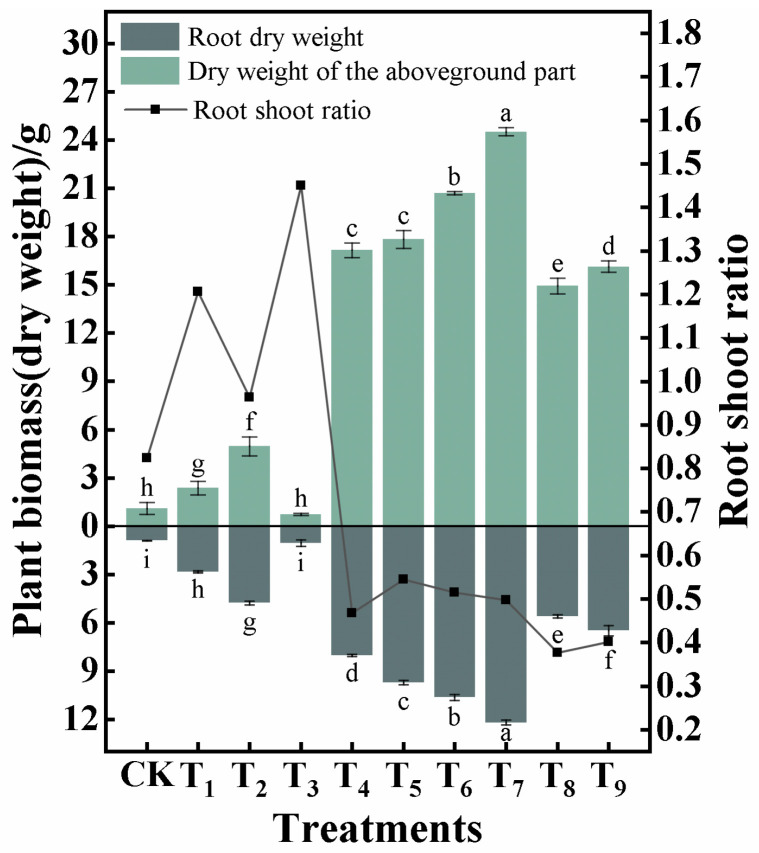
Plant biomass and root–shoot ratio of different treatments. Note: bars represent mean ± standard deviation (SD, n = 3). Different lowercase letters above the bars indicate significant differences among treatments (one-way ANOVA followed by the LSD test, *p* < 0.05).

Figure 6 shows differences in the aboveground growth performance of *A. sparsifolia* among treatment groups, and the normalized growth parameters are presented in Figure 7. The biomass (dry weight) of different plant organs was quantified, and the root–shoot ratio was calculated using Equation (8) [19], with the results shown in Figure 8.

(8) $$R_{r-s}=W_{u}\;/\;W_{0}$$

where $R_{r-s}$ is the root–shoot ratio; $W_{u}$ is the dry weight of the underground part (g); $W_{o}$ is the dry weight of the aboveground part (g).

The measured aboveground growth parameters of *A. sparsifolia* (plant height, crown length, crown width, basal diameter, number of shoot branches, and number of leaves) were normalized. As shown in Figure 6 and Figure 7, the overall growth performance was markedly better in T_4_–T_9_ than in the other treatments. Compared with CK, plant height increased most in T_6_, followed by T_2_, T_5_, and T_7_ (normalized values: 0.65–0.71), whereas T_1_ and T_3_ showed relatively limited increases. Crown length and crown width, as well as basal diameter, were most pronounced in T_6_ and T_7_, with normalized values reaching 0.87–1.00. Relatively higher normalized values for shoot branches and leaves were observed in T_4_ and T_7_. Overall, the amendment combinations in the T_4_–T_9_ treatment groups improved the aboveground growth of *A. sparsifolia*, whereas CK, T_1_, and T_3_ remained at relatively low levels, indicating a weaker growth response to the amendments. These growth responses reflect the ecological performance of the substrate in supporting the growth of *A. sparsifolia*.

As shown in Figure 8, different treatments significantly affected biomass accumulation and allocation. The total biomass of CK, T_1_, T_2_, and T_3_ remained relatively low, among which T_3_ exhibited the poorest growth performance, with total biomass decreasing by 10.40% compared with CK. Except for T_3_, all other treatments increased total biomass (3.21–34.73 g). Amendment combinations also altered the biomass allocation strategies. The root–shoot ratios of CK, T_1_–T_3_ were relatively high, and those of T_1_ and T_3_ exceeded 1.0, indicating greater allocation to roots. In contrast, the root–shoot ratios of T_4_–T_9_ were relatively lower, suggesting increased aboveground allocation and enhanced total biomass accumulation. Across the tested treatments, treatment groups with lower total biomass exhibited higher root–shoot ratios, reflecting the adaptive strategies of *A. sparsifolia* under different substrate conditions.

### 3.4. Root Morphological Distribution Characteristics of A. sparsifolia Under Different Amendment Contents

**Figure 9:**
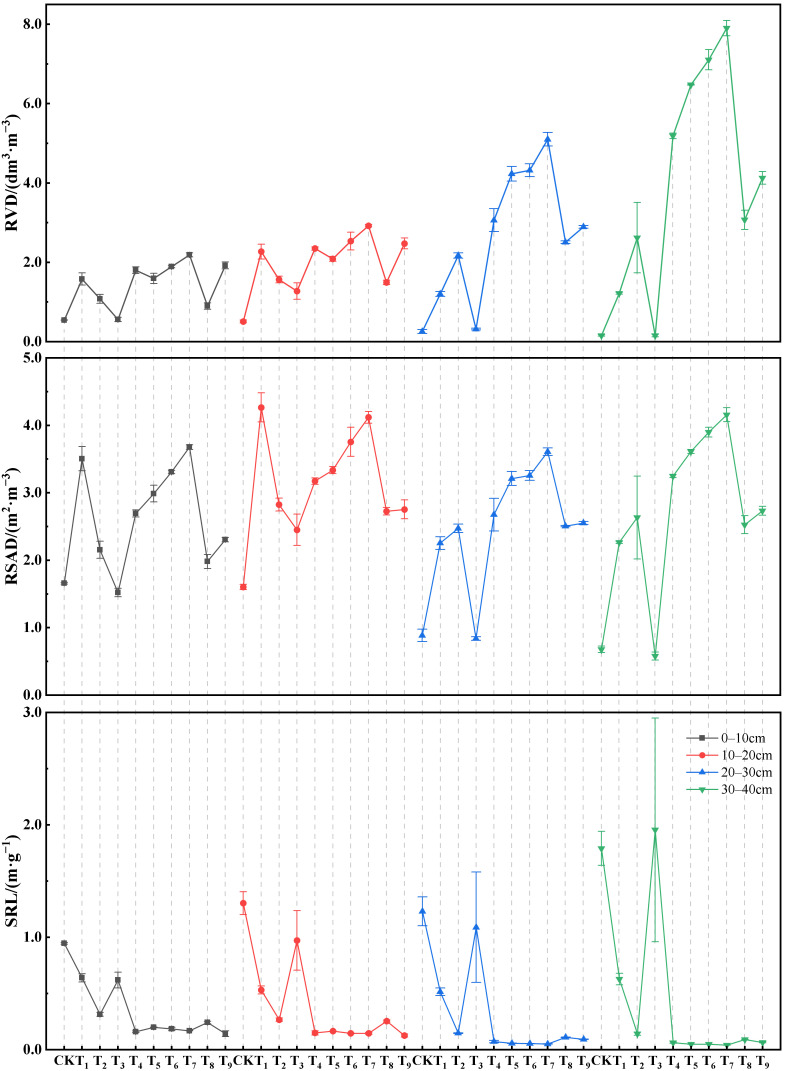
Root morphological distribution characteristics of *A. sparsifolia* under different treatments.

Figure 9 shows differences in the root morphological distribution characteristics of *A. sparsifolia* among treatments. It should be noted that *A. sparsifolia* in CK and the T_1_–T_3_ treatments had a large number of lateral roots, and the distinction between the taproot and lateral roots was not obvious, whereas *A. sparsifolia* in the other treatments exhibited a clear taproot structure. The experimental results indicate that, across all treatments, root length in the substrate layer was significantly greater than that in the sandy soil layer. At 0–20 cm soil depth, differences in root length among treatments were greater than those in root diameter. As the soil layer transitioned from the substrate layer to the sandy soil layer, root length decreased sharply in all treatments. The reduction in the number of lateral roots in the sandy soil layer resulted in a decrease in total root length by 40.68–64.59%; except for CK and T_3_, the average root diameter increased by 19.58–236.09%. Consequently, in sandy soils below 20 cm depth, differences in root diameter among treatments became more pronounced.

The SRL values of CK and the T_1_–T_3_ treatments were significantly greater than those of the other treatments. For T_3_, SRL was relatively small in the 0–20 cm surface soil layer, whereas below 20 cm soil depth, a sharp decrease in root dry weight (RM) led to an increase in SRL in deeper soil layers, resulting in large fluctuations in SRL. In contrast, SRL in the T_4_–T_9_ treatments generally decreased with increasing soil depth and showed relatively small variations within the same soil layer. After transitioning from the substrate layer to the sandy soil layer, the root diameter and root length in CK and T_3_ decreased consistently, resulting in significantly higher RSAD and RVD values in the substrate layer than in the sandy soil layer. For the other treatments, during the initial transition from the substrate layer to the sandy soil layer (20–30 cm), the proportional increase in root diameter was smaller than the proportional decrease in root length, causing RSAD to depend more on changes in root length. When roots reached the 30–40 cm soil depth, the proportional increase in root diameter was much greater than the proportional decrease in root length, and RSAD became more dependent on changes in root diameter rather than root length. Consequently, treatments T_2_ and T_4_–T_9_ exhibited a trend in which RSAD first decreased and then increased with increasing soil depth during the transition from the substrate layer to the sandy soil layer. Because changes in RVD mainly depend on root diameter, RVD values of treatments T_2_ and T_4_–T_9_ in the sandy soil layer were higher than those in the substrate layer. Changes in root morphology reflect trait regulation by the substrate environment and provide a biological basis for subsequent variations in root–soil mechanical interactions.

### 3.5. Shear Strength Characteristics of Root–Soil Composites Under Different Amendment Contents

Table 6 lists the shear test results of root–soil composites at four depth intervals (0–10 cm, 10–20 cm, 20–30 cm, and 30–40 cm) under normal loads of 50, 100, and 150 kPa. Overall, the peak shear stress increased with increasing normal load in all treatments, indicating a clear response of root–soil composites to normal loading. Obvious differences in peak shear stress were also observed among treatments. In the substrate layers (0–10 cm and 10–20 cm), T_1_ exhibited the highest peak shear stress under all normal loads, followed by T_7_, while T_8_ consistently showed the lowest values. Except for CK, peak shear stress generally increased with increasing soil depth, indicating that shear resistance was jointly influenced by root distribution characteristics and soil layer structure. In the sandy soil layers (20–30 cm and 30–40 cm), peak shear stress was generally higher than that in the substrate layer. Under all normal loads, T_1_ still exhibited the highest peak shear stress, suggesting that the reinforcement effect of lateral-root-developed root systems remained evident in the sandy layer. Among treatments with well-developed taproot structures, T_7_ showed relatively high peak shear stress, whereas T_8_ exhibited low peak shear stress under all normal loads.

**Figure 10:**
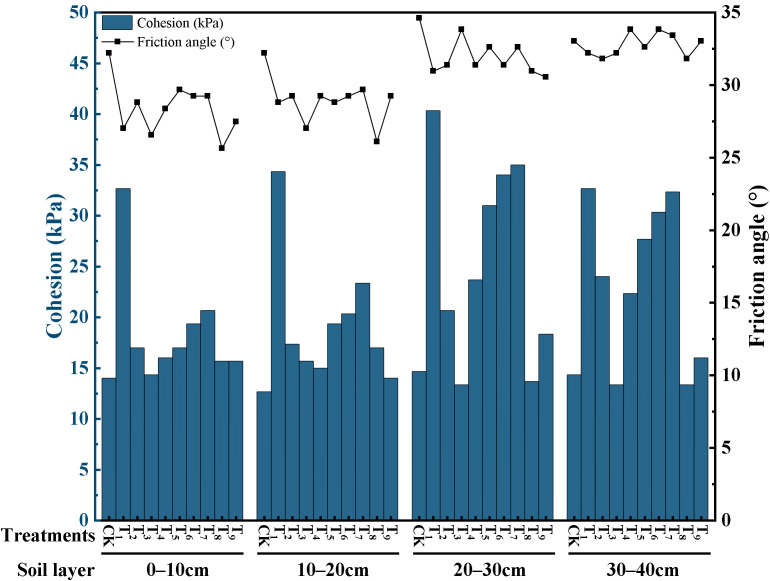
Shear strength indicators of root–soil composite under different treatments.

Figure 10 further illustrates the variations in shear strength parameters (internal friction angle and cohesion) across soil depths. In the substrate layer, CK exhibited a relatively high internal friction angle, whereas its cohesion was significantly lower than that of the amended treatments. The internal friction angle of treatments T_1_–T_9_ ranged from 25.64° to 29.68°, representing decreases of 7.85–20.40% compared with CK. In contrast, cohesion in treatments T_1_–T_9_ increased markedly, with increments ranging from 2.38% to 171.05%. In the 20–40 cm sandy soil layer, differences in internal friction angle among treatments were relatively small, whereas cohesion varied markedly. Compared with CK, cohesion was higher in all amended treatments except T_3_ and T_8_. Among them, T_1_ exhibited the highest cohesion, reaching 40.33 kPa and 32.67 kPa. These results indicate that the enhancement in the mechanical properties of root–soil composites under the amendments was mainly achieved through increased cohesion.

### 3.6. Correlation Analysis and Integrated Eco-Mechanical Effectiveness Evaluation

**Figure 11:**
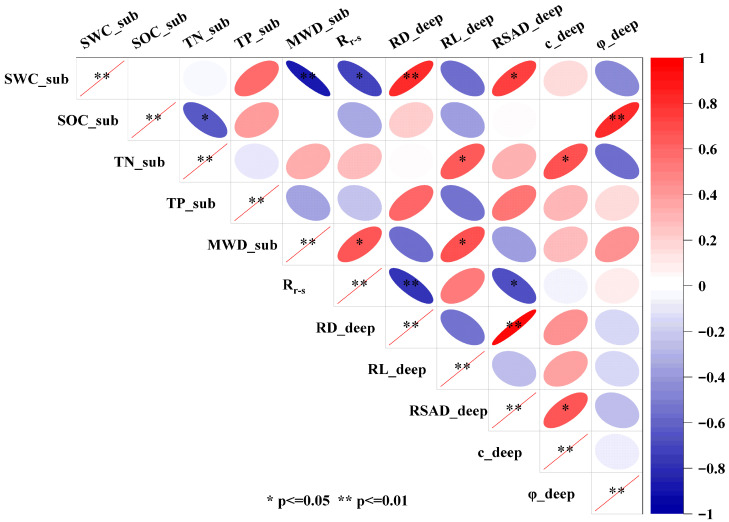
Pearson correlations among sandy substrate properties ($SWC_{sub},SOC_{sub},TN_{sub},TP_{sub},MWD\_sub$), plant traits ($R_{r-s},RD\_deep,$ RL_deep,RSAD_deep), and the shear strength parameters of the deep (sandy) soil layer (∁_deep,φ_deep) under different treatments. Note: SWC_sub, substrate water content (%); SOC_sub, substrate organic carbon content (g·kg^−1^); TN_sub, substrate total nitrogen content (g·kg^−1^); TP_sub, substrate total phosphorus content (g·kg^−1^); MWD_sub, mean weight diameter of substrate aggregates (mm); $R_{r-s}$, Root shoot ratio; RD_deep, root diameter in the deep soil layer; RL_deep, root length in the deep soil layer; RSAD_deep, root surface area density in the deep soil layer; ∁_deep, cohesion of the deep soil layer; φ_deep, cohesion of the deep soil layer. The correlation heatmap was visualized based on treatment mean values. Colors represent correlation coefficients (r). Asterisks indicate significance levels (* *p ≤* 0.05; ** *p ≤* 0.01).

To investigate the correlations among substrate properties, plant traits, and the shear strength of the deep (sandy) soil layer under different treatments, Pearson correlation analysis was performed (Figure 11). Substrate water content (SWC_sub) was strongly and negatively correlated with substrate aggregate stability (MWD_sub) and $R_{r-s}$ (*p* < 0.01), while showing significant positive correlations with deep-layer root diameter (RD_deep) and deep-layer root surface area density (RSAD_deep) (*p* < 0.01). Notably, several root traits exhibited highly coordinated correlation patterns. RD_deep was positively correlated with RSAD_deep (*p* < 0.01), but negatively correlated with deep-layer root length (RL_deep) and $R_{r-s}$ (*p* < 0.05). These relationships indicate a trade-off between root thickening and root extension under different substrate conditions, accompanied by coordinated adjustments in aboveground–belowground biomass allocation and trait expression.

Total nitrogen in the substrate layer (TN_sub) was positively correlated with RL_deep and cohesion (∁_deep) (*p* < 0.05), whereas it was negatively correlated with the internal friction angle (φ_deep) (*p* < 0.05). By contrast, substrate organic carbon content (SOC_sub) showed a strong positive correlation with φ_deep (*p* < 0.01). The results indicate that ∁_deep was closely associated with the coupled regulation of substrate conditions and root development, whereas φ_deep exhibited relatively weaker correlations with most root traits and shows lower sensitivity to deep-root reinforcement.

Based on the correlation structure, six indicators with minimal redundancy (SWC_sub, TN_sub, MWD_sub, RL_deep, RSAD_deep, and ∁_deep) were selected as representative indicators for integrated evaluation. The entropy-weighted TOPSIS method was then used to calculate the integrated eco-mechanical effectiveness index (EMEI) for each treatment. By incorporating material input, a cost-effectiveness index (CEI) was further calculated to identify economically effective substrate formulations. The results are shown in Table 7. T_1_ achieved the highest EMEI value, followed by T_2_ and T_7_, whereas T_3_ ranked last. Although CK showed the highest cost-effectiveness index, its integrated eco-mechanical effectiveness was limited. Overall, T_1_ was identified as the optimal substrate formulation.

## 4. Discussion

### 4.1. Effects of Amendment Contents on Soil Water and Nutrient Characteristics

In arid sandy habitats, low clay content and weak aggregation typically lead to rapid infiltration, high evaporative loss, and poor nutrient retention [51], which together limit plant establishment and subsequent bio-reinforcement of the soil [52,53]. To alleviate these limitations, this study applied composite amendments (CMC, SF, and FA) to improve the sandy substrate habitat for the growth of *A. sparsifolia.*

As the primary amendment component, the addition of CMC significantly increased substrate water content. The rapid water absorption and swelling behavior of CMC was the main driving force for water storage [54], prolonging water residence time in the soil and providing favorable water conditions for plant growth [55]. Mechanistically, CMC hydrogels can not only bind pore water to enhance the water-holding capacity of the substrate and reduce evaporation, but also fill soil pores and block some flow pathways, thereby decreasing gravitational drainage [56]. This explains why higher CMC contents weakened the permeability into the underlying sandy layer, resulting in more water being retained in the 0–20 cm substrate and less water percolating downward. This result is consistent with the findings of Shao, et al. [57], who reported that the excellent water-retention capacity and slow water-release ability of CMC significantly enhanced the water storage capacity of desert soils. In addition, the SF/FA ratio influences the optimal water-holding range by regulating pore structure and the particle skeleton. FA tends to act as a microfiller and reactive mineral component, improving soil structure and properties in terms of strength, deformation resistance, and permeability [58,59]. The incorporation of fibers can fully activate the bridging mechanism to enhance the crack resistance and ductility of concrete [60,61]. However, an excessively high fiber content may increase macroporosity, thereby accelerating evaporation and preferential flow [62]. In this study, the synergistic effect of SF and FA improved infiltration distribution and water storage when the SF/FA ratio was below 1:5. Once SF became excessive (SF/FA > 1:5), macropore formation and cementation disruption became dominant, resulting in accelerated water loss and a decrease in substrate water content. This phenomenon was particularly evident under low to medium CMC contents (0.5% and 1.0%). For the treatments with a high content of CMC (1.5%), the CMC hydrogels formed by water absorption and swelling can effectively block the macropore structures caused by excessive fibers, thereby reducing hydraulic connectivity and suppressing evaporation-induced water loss. This explains why the negative effect of a high SF/FA ratio can be weakened under high CMC conditions. Ning, et al. [63] also experimentally verified the good water storage capacity of CMC and confirmed that it is a suitable water-retention agent for sandy loam soils.

The nutrient distribution patterns can be interpreted as the integrated outcome of two controlling factors: (i) leaching intensity governed by soil water status and pore connectivity, and (ii) biological uptake mediated by root traits. Yin, et al. [64] pointed out that drought restricts solute transport and decomposition in soils. Therefore, in the water-limited substrate soil of this study, SOC content was relatively high, whereas TN and TP contents were low. When the amendments increased water availability, nutrient mobility and plant uptake tended to increase; however, excessive water intensified nutrient leaching losses [31,65]. In treatments with water content exceeding 22.65%, nutrient migration into the underlying sandy layer occurred, resulting in a “first increase and then decrease” response of SOC, TN, and TP along the water-content gradient. For TN, the leguminous nature of *A. sparsifolia* provides an additional pathway via symbiotic nitrogen fixation and rhizosphere processes [66,67]. This implies that TN may be influenced not only by leaching but also by trait-mediated nutrient acquisition strategies [68].

Overall, the composite amendments enhance habitat quality mainly by controlling water retention through CMC and modifying pore-structure-driven water and nutrient transport via the SF/FA combination, which accounts for the proportion-dependent responses observed among treatments.

### 4.2. Effects of Amendments on Soil Aggregate Size Distribution and Stability

Soil aggregates are the basic units of soil structure, and their stability affects soil strength and erosion resistance [69,70]. In addition, overall soil stability is often regarded as a key indicator of ecosystem processes [71]. As shown in Figure 5, large macro-aggregates (>2 mm) and small macro-aggregates (0.25–2 mm) accounted for as much as 92.61% in the CK group, whereas micro-aggregates (0.053–0.25 mm) and silt and clay particles (<0.053 mm) together accounted for only 7.39%. The results indicate that the incorporation of amendments can drive the fragmentation of macro-aggregates. In addition, the swelling and shrinkage stresses of water-retention agents during repeated water absorption and release may also induce swelling-induced cracks and microcracks in the soil, weakening the integrity of the original macro-aggregates and thereby increasing the proportion of fine particles [72]. Huang, et al. [73] used super absorbent resin (SAR) as a water-retention agent and similarly found that SAR disrupted aggregate structure and reduced aggregate stability, making aggregates more prone to fragmentation into smaller aggregates, and confirmed this phenomenon through microscopic analysis (scanning electron microscopy). Meanwhile, unreacted FA may exist in the substrate and act as an inert microfiller to alter soil structure, ultimately leading to an increase in fine soil particles [74]. Together, these processes provide a mechanistic explanation for the decrease in macro-aggregate proportion and the decline in WSA and MWD in the amended substrate.

In addition, the application of organic amendments significantly promoted root growth. Root growth can counteract physicochemical fragmentation by binding particles via compression, enmeshment, and rhizodeposition, thereby promoting aggregation and stabilizing soil structure [75,76]. Notably, the extent to which aggregate stability is affected may largely depend on root functional traits [77]. Numerous studies have confirmed that fine roots play a key role in regulating soil aggregate stability [78]. The T_1_ treatment with well-developed lateral roots exhibited a clear fine-root advantage, in which abundant fine roots increased the contact area and local enmeshment intensity, thereby resulting in good aggregate stability. In contrast, the expansion of coarse roots may induce preferential flow or pore disruption and thus disturb local soil structure [79,80], which may explain the slight decrease in MWD observed in T_7_ with relatively thicker taproots. Accordingly, treatments with distinct root traits showed pronounced differences in aggregate stability, indicating that abundant fine roots are generally more favorable for maintaining soil aggregate stability than coarse-root-dominated systems. Similar results were reported by Li, et al. [81], who found that the rhizosphere of fibrous-root plants played a significant role in promoting soil aggregate diameter, whereas the rhizosphere of taproot plants had no significant influence on the diameter of aggregate.

### 4.3. Interactions Between Soil Properties and Plant Growth Characteristics: Mechanistic Explanation of the “Adaptive Strategy”

The composite amendments altered the water and nutrient distribution and structural conditions of the growth habitat of *A. sparsifolia*, and *A. sparsifolia* adapted to different habitats by reallocating biomass and modifying root architecture to achieve resource acquisition.

Under resource-poor or drought-prone conditions, an increase in the root–shoot ratio represents an adaptive strategy of vegetation [82], allowing resources (dry matter) to be allocated to roots [83], thereby acquiring resources through an extensive root system and performing important ecological functions. In this study, the higher root–shoot ratios in CK and T_1_–T_3_ likewise reflect the resource allocation tendency of plants under infertile or water-limited conditions, and while biomass is shifted to the root system, total biomass also decreases. In contrast, the amended treatments T_4_–T_9_ provided more sufficient water and nutrient conditions, enabling plants to allocate more dry matter to aboveground parts and increase overall biomass, indicating that habitat quality was improved and the allocation demand for stress avoidance was reduced [84]. In addition, this study found that both excessive water and drought stress can limit the growth of *A. sparsifolia*. Under high soil water content, nutrient leaching and reduced oxygen supply can constrain root function, hydraulic conductivity, and nutrient uptake efficiency, thereby reducing biomass [35]. Therefore, decreased plant biomass was observed in the T_4_, T_8_, and T_9_ treatment groups under excessive water conditions.

Root traits link ecology and mechanics [85]. In this study, root traits exhibited diverse response to the soil habitat changes caused by the composite amendments. The results showed that under moderate habitat stress, *A. sparsifolia* tended to enhance lateral root expansion and fine-root proliferation. This dense root system increased the contact density between roots and soil particles, facilitating root anchorage and penetration into deeper and denser soils while strengthening root–substrate interactions. In addition, apart from intrinsic plant plasticity, the differences in soil properties and root development among treatments may also be related to plant–microbe interactions, which are crucial for plant growth and adaptation to desert environments. Yang, et al. [86] reported that *A. sparsifolia*, as a dominant vegetation species and a key mycorrhizal plant in desert riparian systems, can improve and protect soil structure through mycorrhizal symbiosis, and can also enhance host water and nutrient uptake efficiency via widely distributed hyphae, thereby improving plant stress resistance [87,88]. It has been reported that the colonization rate of AMF in *A. sparsifolia* can reach 90%, with colonization intensity up to 60% [20]. In the present study, the synergistic effects of CMC, SF, and FA altered the substrate environment and root structure, which may theoretically affect AMF colonization and activity, thereby indirectly regulating plant growth and nutrient utilization. In particular, previous studies have also confirmed that diverse fungal communities exist in the roots of *A. sparsifolia*, and their community structures differ among growth stages [89].

The above root trait variations provide a mechanistic basis for the “adaptive strategy” claim, whereby *A. sparsifolia* balances resource acquisition efficiency and environmental constraints by adjusting biomass allocation and root architecture. This coordination, in turn, influences soil structural development and the mechanical performance of root–soil interactions. The correlation analysis (Figure 11) further supports the interpretation that adaptive changes in root traits mediate the reinforcement process, thereby contributing to enhanced soil strength.

### 4.4. Effects of Soil Properties and Root Morphology on the Shear Strength of Root–Soil Composites: From Mechanisms to Engineering Implications

The shear strength of the sandy substrate reflects the combined effects of substrate strength and root reinforcement. The amended substrate improved its structure through swelling–shrinkage, microfilling, and bridging effects; optimized water and nutrient conditions; and promoted root development, thereby enhancing shear strength.

For CK, the substrate environment was unfavorable for plant growth and could not support root penetration into the soil layers or maintain sustained root–soil contact, which limited root–soil interface interactions and resulted in an insufficient cohesion contribution. Meanwhile, an excessively high soil water status was also a key factor limiting the shear strength of the substrate [90]. Beyond substrate conditions, root traits markedly influence the mechanisms by which roots contribute to soil shear strength [91], particularly in the sandy soil layer where regulation capacity is weaker. The correlation analysis further indicates that substrate nutrient status and root morphological traits are significantly associated with cohesion in the deep sandy soil layer. By contrast, the internal friction angle shows weaker correlations with most root traits, suggesting that biological regulation enhances shear strength mainly through cohesion-dominated mechanisms. Root systems with well-developed lateral roots and higher fine-root density or root surface area provide more bridging points across the shear plane and a more uniformly distributed reinforcement effect, thereby producing a greater increase in cohesion relative to CK. In contrast, when lateral roots are sparse and the root system is dominated by thick taproots, reinforcement relies more on interface friction and local interlocking, and thus the increase in cohesion may remain limited even when root diameter is large.

When shear deformation occurs, roots crossing the shear zone mobilize tensile resistance, thereby restraining the relative displacement of soil particles [92]. Because fine roots have a high number density and a wide spatial coverage, this mechanism is particularly effective for dense fine-root networks [93]. In treatments and soil layers dominated by taproots, the observed surface abrasion of roots without breakage indicates that frictional interactions at the root–soil interface and confinement-driven interlocking contribute substantially to resistance, and this effect is more strongly correlated with root surface area density [94]. Therefore, some taproot-dominated treatments can exhibit relatively high shear strength due to the high degree of root–soil contact, whereas a large root diameter without sufficient lateral roots does not guarantee high shear strength, as exemplified by the T_4_ and T_9_ treatments.

Because shallow landslides and shear failure are the primary failure modes of ecological substrates [24,95], enhancing bonding strength through optimization of fine-root networks is of practical significance, while sustained root penetration can improve deep anchorage. This study proposes a trait-based regulation pathway in an engineered sandy substrate, in which composite amendments reshape the vertical water and nutrient niches and induce coordinated plasticity of root traits, thereby altering soil structural properties and the shear resistance between roots and soil within a unified framework. It should be clearly noted that this study has the following limitations: (i) the experiment covered only one growing season, and over longer time scales, the aging and degradation processes of the composite amendments may continuously alter soil structure and hydrological properties, while the coupled relationship between the deterioration rate of the amendments and the growth rate of root reinforcement capacity remains unclear; (ii) this study focused on a single desert species (*A. sparsifolia*), and therefore the generalizability of the findings to other desert vegetation types and their distinct root trait strategies requires further validation; (iii) this study did not quantitatively characterize the interactions between roots and soil microorganisms (e.g., mycorrhizae), and therefore the biological regulatory effects are currently inferred rather than directly measured.

## 5. Conclusions

(1) This study supports the hypothesis that composite amendments (CMC–SF–FA) can improve the habitat quality of sandy substrate by regulating soil water and nutrient distribution in a proportion-dependent manner. CMC primarily enhanced near-surface water storage, whereas the SF/FA ratio regulated pore structure and thus controlled water and nutrient transport. Nutrient retention exhibited a threshold response, with excessive water content leading to increased nutrient leaching into the sandy layer.
(2) The amendments reorganized soil aggregate size distribution and structural stability, suggesting that amendment-driven swelling–shrinkage, microfilling processes, and bridging effects jointly modified soil physical structure, thereby creating favorable conditions for root penetration and development.
(3) The results demonstrate that root functional traits constitute the mechanistic link between ecological improvement and mechanical reinforcement. Amendment-driven habitat shifts triggered coordinated plasticity in biomass allocation and root architecture. Under moderate habitat stress, *A. sparsifolia* tended to promote lateral-root expansion and fine-root proliferation, increasing root–soil contact density and strengthening root–substrate interactions, thereby enhancing the shear strength of root–soil composites. These findings highlight the novelty of a trait-based substrate optimization strategy for engineered sandy substrates prone to shallow shear failure.
(4) Among the tested combinations, the T_1_ configuration exhibited the best coupling between plant establishment and mechanical reinforcement, indicating that proportion-optimized amendment design can achieve coordinated enhancement of ecological suitability and shear strength. Based on the integrated eco-mechanical effectiveness and cost-effectiveness evaluation, T1 was identified as the optimal substrate formulation. However, these conclusions are constrained by the short study duration (one growing season) and the use of a single desert species (*A. sparsifolia*). Therefore, the identified optimal proportion and the proposed trait-based pathway should be interpreted as species- and growth-stage-specific, and require validation across other desert vegetation types and longer time scales before broad generalization.

## Figures and Tables

**Table 1:** Materials used for preparing the sandy substrate: sources and key properties.

Material	Source	Key Properties/Specification	Preparation/Notes
Planting soil	slope near the Manas River in Xinjiang, China (43°59′ N, 85°57′ E)	Natural dry density: 1.58 g/cm^3^; classified as clayey sand (USCS): 12.7% clay, 38.3% silt, 41.3% sand, and 7.7% gravel.	Air-dried, impurities moved, and sieved through an 8 mm mesh according to (NB/T 35082, 2016) [38]
Cement	Xinjiang Tianshan Cement Co., Ltd., Urumqi, China	Low-alkali sulfoaluminate cement	/
Peat soil (organic matter)	Hebei Shijiazhuang Wu Zhanggui Horticulture Co., Ltd., Hebei, China	pH = 5.5–6.0	/
Slow-release fertilizer	Xinjiang Shihezi Flower Market, Shihezi, China	Nutrient composition ratio (N:P_2_O_5_:K_2_O) = 14:13:13	/
Water retention agent (carboxymethyl cellulose sodium, CMC)	Xinjiang Shihezi Chemical Factory, Shihezi, China	Non-ionic water-soluble gel	/
Straw fibers (SF)	Field straw (locally collected)	Toughness and extensibility	Uniformly crushed to 1–2 cm in length to ensure substrate homogeneity
Fly ash (FA)	Henan Word Mei Environmental Technology Co., Ltd., Hean, China	F-class 1; rich in Ca, S, Fe, Mn, Si, and other beneficial trace elements for plant growth	/

**Table 2:** Level table of testing factors.

Levels	CMC (%)	SF (%)	FA (%)
1	0.5	1	5
2	1	1.5	10
3	1.5	2	20

**Table 3:** Orthogonal design of surface substrates.

Substrate No.	Orthogonal Design Matrix			
	CMC (%)	SF (%)	FA (%)	CK (%)
T_1_	0.5	1	5	0
T_2_	0.5	1.5	10	0
T_3_	0.5	2	20	0
T_4_	1	1	10	0
T_5_	1	1.5	20	0
T_6_	1	2	5	0
T_7_	1.5	1	20	0
T_8_	1.5	1.5	5	0
T_9_	1.5	2	10	0

Note: CK, T_1_–T_9_ represent the control group and 9 experimental groups, respectively; the amount of all materials refers to the ratio of the material mass to the dry mass of the soil.

**Table 4:** Experimental scheme and design.

Sample Type	Parameter	Value (s)	Remarks
Plant aboveground parts	Sample quantity	30	Each treatment group selected 3 samples for measuring plant growth indicators
Undisturbed soil samples	Sample size (cm^3^)	5 × 5 (diameter × height)	Each treatment group collected 3 soil samples of the same soil type for the determination of soil aggregate stability and soil nutrient content
	Sampling depth (cm)	0–20 (substrate layer), 20–40 (sandy soil layer)	
	Sample quantity	180	
Undisturbed root–soil composite samples	Sample size (cm^3^)	10 × 10 × 8.5 (length × width × height)	Each treatment group prepared 3 root–soil composite shear samples at the same soil depth
	Sampling depth (cm)	0–10, 10–20, 20–30, 30–40	
	Sample quantity	120	

**Table 5:** Characteristics of soil aggregate stability parameters at depths of 0–20 cm and 20–40 cm.

Treatments	Soil Layer (cm)	WSA (%)	MWD (mm)
CK	0–20	92.61 ± 1.10 ^a^	1.60 ± 0.02 ^a^
T_1_	0–20	88.98 ± 0.28 ^b^	1.53 ± 0.01 ^b^
T_2_	0–20	86.77 ± 1.03 ^c^	1.45 ± 0.01 ^c^
T_3_	0–20	85.96 ± 1.25 ^cd^	1.39 ± 0.01 ^d^
T_4_	0–20	80.53 ± 0.59 ^f^	1.18 ± 0.01 ^h^
T_5_	0–20	83.20 ± 0.68 ^e^	1.32 ± 0.01 ^g^
T_6_	0–20	84.96 ± 0.41 ^d^	1.37 ± 0.01 ^e^
T_7_	0–20	84.90 ± 0.57 ^d^	1.34 ± 0.01 ^f^
T_8_	0–20	76.07 ± 0.75 ^h^	1.07 ± 0.01 ^j^
T_9_	0–20	77.59 ± 0.98 ^g^	1.10 ± 0.01 ^i^
CK	20–40	60.43 ± 1.54 ^def^	0.96 ± 0.03 ^de^
T_1_	20–40	68.95 ± 0.98 ^a^	1.13 ± 0.02 ^a^
T_2_	20–40	59.36 ± 1.04 ^efg^	0.96 ± 0.01 ^de^
T_3_	20–40	58.54 ± 0.72 ^g^	0.93 ± 0.01 ^ef^
T_4_	20–40	61.54 ± 0.53 ^d^	1.03 ± 0.01 ^c^
T_5_	20–40	65.02 ± 1.17 ^c^	1.06 ± 0.02 ^b^
T_6_	20–40	66.35 ± 0.36 ^bc^	1.09 ± 0.01 ^b^
T_7_	20–40	67.01 ±1.03 ^b^	1.07 ± 0.01 ^b^
T_8_	20–40	58.73 ± 0.58 ^fg^	0.92 ± 0.01 ^f^
T_9_	20–40	60.79 ± 1.41 ^de^	0.97 ± 0.03 ^d^

Note: Values are means ± standard deviation (SD, n = 3). Different lowercase letters indicate significant differences in soil aggregate stability parameters (WSA and MWD) within the same soil layer among different treatments (one-way ANOVA followed by the LSD test, *p* < 0.05).

**Table 6:** Shear strength of the root–soil composite under different treatments.

Treatments	0–10 cm			10–20 cm			20–30 cm			30–40 cm		
	50 kPa	100 kPa	150 kPa	50 kPa	100 kPa	150 kPa	50 kPa	100 kPa	150 kPa	50 kPa	100 kPa	150 kPa
CK	45	78	108	44	76	107	48	86	117	47	79	112
T_1_	57	86	108	61	91	116	77	99	131	66	92	129
T_2_	44	73	99	45	74	101	50	84	111	54	88	116
T_3_	39	65	89	40	69	91	47	80	114	46	74	109
T_4_	43	70	97	43	71	99	54	85	115	56	89	123
T_5_	45	75	102	47	74	102	62	97	127	60	91	124
T_6_	47	76	103	48	77	104	63	98	124	63	99	130
T_7_	48	78	104	51	82	108	67	99	131	64	100	130
T_8_	39	65	87	41	67	90	44	73	104	45	74	107
T_9_	41	69	93	42	70	98	47	79	106	48	82	113

**Table 7:** Integrated eco-mechanical effectiveness and cost-effectiveness of different substrate formulations.

Treatments	Eco-Mechanical Effectiveness Index (EMEI)	Rank of EMEI	Cost Index	Cost-Effectiveness Index (CEI)	Rank of CEI
CK	0.375	5	1	0.375	1
T_1_	0.888	1	7.5	0.118	2
T_2_	0.448	2	13	0.034	4
T_3_	0.155	10	23.5	0.007	10
T_4_	0.228	7	13	0.018	6
T_5_	0.334	6	23.5	0.014	8
T_6_	0.383	4	9	0.043	3
T_7_	0.410	3	23.5	0.017	7
T_8_	0.190	8	9	0.021	5
T_9_	0.180	9	14.5	0.012	9

Note: The eco-mechanical effectiveness index (EMEI) is the relative closeness coefficient (C) derived from the entropy-weighted TOPSIS method; the cost index is based on the total dosage of CMC, SF, and FA (CK = 1); the cost-effectiveness index (CEI) was calculated as EMEI divided by the cost index.

## Data Availability

The original contributions presented in this study are included in the article. Further inquiries can be directed to the corresponding author.
